# Kiamycin, a Unique Cytotoxic Angucyclinone Derivative from a Marine *Streptomyces* sp.

**DOI:** 10.3390/md10030551

**Published:** 2012-02-27

**Authors:** Zeping Xie, Bing Liu, Hongpeng Wang, Shengxiang Yang, Hongyu Zhang, Yipeng Wang, Naiyun Ji, Song Qin, Hartmut Laatsch

**Affiliations:** 1 Yantai Institute of Coastal Zone Research, Chinese Academy of Sciences, Yantai 264003, China; Email: zpxie@yic.ac.cn (Z.X.); bliu@yic.ac.cn (B.L.); hyzhang@yic.ac.cn (H.Z.); ypwang@yic.ac.cn (Y.W.); nyji@yic.ac.cn (N.J.); 2 Graduate University of Chinese Academy of Sciences, Beijing 100049, China; 3 Department of Organic and Biomolecular Chemistry, University of Göttingen, Göttingen D-37077, Germany; Email: wanghongpeng@hotmail.com; 4 Chemical Biological Research Institute, College of Science, Northwest A & F University, Yangling 712100, China; Email: ysx19821028@yahoo.com.cn

**Keywords:** angucyclinone, epoxybenz[a]anthracene, marine *Streptomyces*, cytotoxicity

## Abstract

Kiamycin (**1**), a new angucyclinone derivative possessing an 1,12-epoxybenz[a]anthracene ring system, was isolated from the marine *Streptomyces* sp. strain M268 along with the known compounds 8-*O*-methyltetrangomycin (**3**) and 8-*O*-methylrabelomycin (**4**). Their structures were elucidated by detailed spectroscopic analysis and comparison with literature data. The new angucyclinone derivative showed inhibitory activities against the human cell lines HL-60 (leukemia), A549 (lung adenocarcinoma), and BEL-7402 (hepatoma) with inhibition rates of 68.2%, 55.9%, and 31.7%, respectively, at 100 µM. It appears to have potential as an anticancer agent with selective activity.

## 1. Introduction

Angucyclinones, benz[a]anthraquinone derivatives isolated mainly from *Streptomyces* spp. [1], have attracted much attention due to their broad and strong biological activities, mainly as antiviral, antifungal, anti-tumor and enzyme inhibitory drugs [2,3]. Up to now, more than 100 angucyclinones have been described since the discovery of tetrangomycin and tetrangulol in 1965 [3,4]. However, less than 20 of them possess epoxybenz[a]anthraxcene structures, and most of the latter are 6a,12a-epoxides. Among the others, gephyromycin (**2**) is of some interest because of its remarkable structure. It was isolated from a *Streptomyces* sp. by Bringmann *et al*. in 2005 and was found to exhibit glutaminergic activity on neuronal cells [5]. 

**Figure 1:**
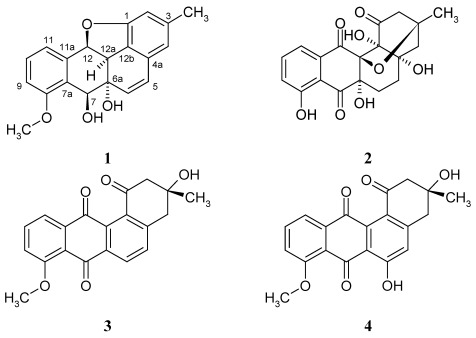
Structures of kiamycin (**1**); gephyromycin (**2**); 8-*O*-methyltetrangomycin (**3**); and 8-*O*-methylrabelomycin (**4**).

In our investigation of marine microbes in Kiaochow Bay of China, a *Streptomyces* strain M268 yielded a further unique angucyclinone derivative, a 1,12-epoxybenz[a]anthracene (**1**). In this paper, we report the isolation, structure elucidation and cytotoxicity of compound **1** (Figure 1) against the human cell lines HL-60 (leukemia), BEL-7402 (hepatoma), and A549 (lung adenocarcinoma).

## 2. Results and Discussion

### 2.1. Isolation and Structure Elucidation of Kiamycin

The streptomycete strain M268 was isolated from a marine sediment sample collected near Qingdao and cultivated on M_2_^+^ medium with aged seawater. After 7 days at 28 °C, the culture filtrate and the mycelium were extracted with ethyl acetate, and the combined extracts were separated by silica gel column chromatography. The nonpolar first fraction was further purified by column chromatography on Sephadex LH-20 and reversed phase RP-18, and by preparative TLC to deliver the colorless UV absorbing **1** and two orange angucyclinones **3** and **4**.

Compound **1** was obtained as colorless powder with a negative sign of optical rotation (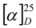
= –113.9; *c* 0.01, MeOH); the molecular formula was established as C_20_H_18_O_4_ by HRESIMS of the pseudomolecular ion peak at *m/z* 345.1103 [M + Na]^+^ (calcd for C_20_H_18_O_4_Na: 345.1097). The ^1^H NMR and COSY spectra indicated a 1,2,3-trisubstituted benzene ring by signals of *o*-coupled protons at δ 6.85 (d), 7.18 (d), and 7.38 (t); the expected *m*-coupling was not visible but was indicated by a line broadening and was visible in the COSY spectrum. A second benzene unit was connected with a methyl group (δ 2.24, s), which was flanked by two protons (broadened singlets at δ 6.41 and 6.50), as indicated by long-range couplings among each other and with the methyl group, respectively, and by NOE correlations with 3-Me. The 9.5 Hz coupling constant of two further doublets at δ 5.88 and 6.74 indicated a *cis*-configured double bond. Finally, a methoxy singlet appeared at δ 3.80, and signals of three oxymethine protons were seen at δ 4.11 (d), 4.85 (d), and 6.00 (d); the residual two hydrogen atoms should belong to hydroxy groups. 

The ^13^C NMR and DEPT spectra of **1** revealed the expected 20 carbon signals, which were classified as two methyls, ten methines, and one oxygenated tertiary carbon (δ 75.8); the remaining seven signals belonged to aromatic tertiary carbons (Table 1). As the molecular formula required 12 double bond equivalents, together with the partial structures mentioned above, compound **1** must be pentacyclic.

**Table 1:** ^1^H, ^13^C, Correlated Spectroscopy (COSY) and Heteronuclear Multiple Bond Correlation (HMBC) NMR data of kiamycin (**1**) in CDCl_3_.

Position	δ_C_, assignm.	δ_H_, mult. ( *J* in Hz)	^1^H–^1^H COSY	HMBC
1	156.7, C_q_			
2	110.5, CH	6.41, s br	H-4, 3-CH_3_	C-1, C-4, C-12b, 3-CH_3_
3	140.1, C_q_			
4	117.6, CH	6.50, s br	H-2, 3-CH_3_	C-2, C-5, C-12b, 3-CH_3_
4a	131.5, C_q_			
5	130.0, CH	6.74, d (9.5)	H-6	C-4, C-4a, C-6a, C-12b
6	134.9, CH	5.88, d (9.5)	H-5	C-4a, C-12a
6a	75.8, C_q_			
7	66.4, CH	4.85, d (1.5)	H-12a	C-6a, C-7a, C-8, C-11a, C-12a
7a	125.5, C_q_			
8	158.3, C_q_			
9	111.0, CH	6.85, d (8.3)	H-10, H-11	C-7a, C-8, C-11
10	130.1, CH	7.38, t (7.9)	H-9, H-11	C-8, C-11a
11	123.3, CH	7.18, d (7.9)	H-9, H-10	C-7a, C-9, C-12
11a	133.0, C_q_			
12	82.6, CH	6.00, d (10.4)	H-12a	C-1, C-6a, C-7a, C-11, C-11a, C-12a, C-12b
12a	44.0, CH	4.11, d (10.4)	H-7, H-12	C-6a, C-7, C-11a, C-12b
12b	122.0, C_q_			
8-OCH_3_	55.9, CH_3_	3.80, s		C-8
3-CH_3_	22.0, CH_3_	2.24, s	H-2, H-4	C-2, C-3, C-4

**Figure 2:**
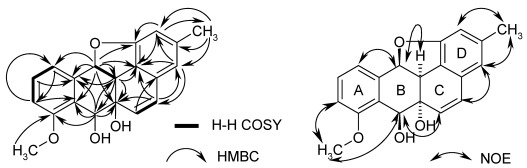
HMBC and COSY correlations (left) and NOESY correlations (right) of kiamycin (**1**).

Both the methoxy signal and the triplet of H-10 showed HMBC couplings with the same carbon at δ 158.3, which assigned the methoxy group to C-8. Further HMBC signals (see Figure 2) with key correlations from H-12 to C-11 (and *vice versa*), and from H-7 to C-8 resulted in an oxygenated tetraline sub-structure (rings A and B in **1**). Cross signals of the double bond protons extended this system by ring C and allowed the attachment of ring D only in an angular manner, resulting in the carbon skeleton of a non-quinonoid anthracyclinone derivative. The positions of the substituents were additionally confirmed by NOE contacts between 3-Me and H-2/4, by correlations of OMe with H-7 and H-9, and of H-6 with H-7. This agrees well with 8-*O*-methyltetrangomycin (**3**) and 8-*O-*methylrabelomycin (**4**), isolated from the same strain in parallel. The structures of the latter were elucidated by spectral data and comparison with literature values [6].

**Figure 3:**
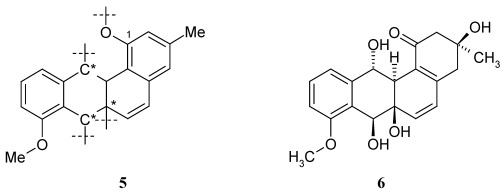
Partial structure **5** of kiamycin (**1**) with four open valences; C* = oxygenated carbon; **6** = X-14881F.

Four oxygenated carbon atoms (C-1, 6a, 7, 12) still had open bonds (partial structure **5** in Figure 3). As only three oxygen atoms were left and only 2 OH groups can be expected according to the proton number, the residual double bond equivalent must be due to a ring closure between two of these carbons via an oxygen bridge, while the OH groups are connected with the remaining two downfield carbons: Rings could be formed between C-1/12 (6a,7-OH), C-6a/7 (1,12-OH), C-6a/12 (1,7-OH), and C-7/12 (1,6a-OH), where the numbers in brackets indicate the position of the additional OH groups. It is obvious that in three of these cases a phenol is formed at C-1, which should easily be methylated with diazomethane. However, as no methyl ether was formed under standard conditions, **1** is the only remaining plausible structure.

The relative configuration of **1** was tentatively derived from coupling constants and NOE correlations: The large coupling constant (9.5 Hz) between H-12 and H-12a and the strong NOE between these atoms agrees best with a dihedral angle close to 0° (*cis*-configuration), which requires a *trans*-orientation of the furan C-12/O bond. Protons H-7 and H-12a are showing a strong W-coupling (*J* = 1.6 Hz), which requires a coplanar orientation of the connecting bonds. This is achieved only if rings B and C are also *cis*-fused; in agreement with the experiment, no NOE is expected between H-7 and H-12 or H-12a in this configuration, as H-7 is too far away. Compound **1** is therefore *rel*-(5a*R*,6*R*,10b*R*,11c*S*)-7-methoxy-2-methyl-10b,11c-dihydro-6*H*-11-oxa-benzo[bc]aceanthrylene-5a,6-diol, which we suggest to name kiamycin. For better comparison, we are using the conventional angucycline numbering in this paper.

**Figure 4:**
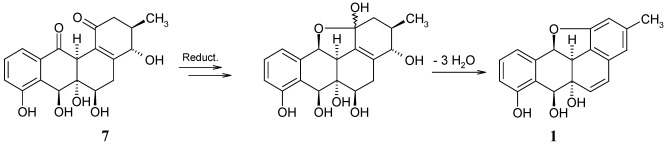
Hypothetical biotransformation of panglimycin B (**7**) into kiamycin (**1**).

The biosynthesis of **1** has not yet been investigated. A plausible approach is, however, the cyclisation of a precursor like saccharithrixin C, elmycin E, or the recently isolated X-14881F (**6**) [7], and the successive loss of water. While these precursors might end up with a wrong configuration of **1**, panglimycin B (**7**) [8] should deliver the correct stereochemistry (Figure 4).

To date, eight natural epoxybenz[a]anthracene derivatives have been described, seven of which were isolated from *Streptomyces* spp. [5,8,9,10,11,12]. None of them has an 11-oxabenzo[bc]aceanthrylene skeleton like **1**. However, a similar benzofuran was formed easily as an intermediate in the synthesis of hatomarubigin [13].

### 2.2. Biological Activity

In the cytotoxicity tests, kiamycin (**1**) exhibited activity against human cell lines, namely HL-60 (leukemia), lung adenocarcinoma cells A549, and the hepatoma cell line BEL-7402 with inhibition values of 68.2%, 55.9%, and 31.7% at 100 μM; adriamycin as positive control showed inhibition rates of 70.0%, 79.3%, and 80.0%, respectively (Table 2). Both compounds were equivalently cytotoxicity against HL-60, while **1** had significantly weaker inhibitory effects against A549 and BEL-7402, as indication of a selective cytotoxicity.

**Table 2:** Cytotoxicity of kiamycin (**1**) and adriamycin against human cell lines HL-60 (leukemia), BEL-7402 (hepatoma), and A549 (lung adenocarcinoma) at 10^−4^ M.

Cancer Cell Line	Inhibition Rate by 1 (%)	Inhibition Rate by Adriamycin (%)
human leukemic cell line HL-60	68.8	70.0
human hepatoma cell line BEL-7402	31.7	79.3
human lung adenocarcinoma cell line A549	55.9	80.8

## 3. Experimental Section

### 3.1. General

Optical rotations were determined with a Perkin-Elmer polarimeter (model 243). ^1^H NMR spectra were recorded on a Varian Inova 300 spectrometer at 300 MHz, ^13^C NMR spectra were recorded with a Bruker Avance 500 spectrometer at 125.7 MHz. Chemical shifts were measured relatively to tetramethylsilane (TMS) as internal standard. 2D NMR spectra: H,H COSY spectra (^1^H,^1^H Correlated Spectroscopy), HMBC spectra (Heteronuclear Multiple Bond Correlation), HSQC spectra (Heteronuclear Single Quantum Coherence) and NOESY spectra (Nuclear Overhauser Effect Spectroscopy). ESI MS was recorded on a Finnigan LCQ with quaternary pump Rheos 4000 (Flux Instrument). ESI HRMS was performed on a Micromass LCT mass spectrometer coupled with a HP1100 HPLC with a diode array detector, or on an Apex IV 7 Tesla Fourier-Transform Ion Cyclotron Resonance Mass Spectrometer (Bruker Daltonics, Billerica, MA, USA). 

Sephadex LH-20 (Amersham Biosciences, Uppsala, Sweden), ODS-A (YMS, 100A, 50 µm), silica gel (Merck) 60-120 mesh for column chromatography, and pre-coated TLC sheets (layer thickness 0.2 mm) and preparative TLC plate (layer thickness 1.25 mm) of silica gel 60 GF_254_ were used. Spots were detected on TLC under UV light or by heating after spraying with 5% H_2_SO_4_ in methanol.

### 3.2. Isolation of the Producing Strain

The marine *Streptomyces* sp. M268 was isolated on Gause’s synthetic agar medium (soluble starch 20 g, KNO_3_ 1 g, K_2_HPO_4_ 0.5 g, MgSO_4_·7H_2_O 0.5 g, FeSO_4_·7H_2_O 0.01 g, K_2_Cr_2_O_7_ 0.3 g, seawater 500 mL, deionized water 500 mL, pH 7.4) from marine sediments of Kiaochow Bay, Qingdao. The strain is preserved within the biological resources department, Yantai Institute of Coastal Zone Research, Chinese Academy of Sciences. 

### 3.3. Fermentation and Isolation

A pre-culture grown on M_2_^+^ agar medium (malt extract 10 g, yeast extract 4 g, anhydrous glucose 4 g, agar 15 g, deionized water 500 mL, seawater 500 mL) at 28 °C for 2 days was used to inoculate 20 L of M_2_^+^ medium. The fermentation was performed in 1 L Erlenmeyer flasks (250 mL broth each) on a linear shaker (120 rpm) for 7 days at 28 °C.

The culture broth was filtered to separate mycelium and water phase. The water phase was extracted with ethyl acetate. The mycelium was dried at 50 °C, and extracted three times with ethyl acetate under ultrasonic radiation. Both extracts were combined, dissolved in methanol and defatted with cyclohexane. The evaporation residue (3.7 g) from the MeOH layer was separated by CC on silica gel with a CH_2_Cl_2_-MeOH (100:0→50:50) gradient to provide seven fractions (Fr.1-7). Fr.1 (0.26 g) was further purified by Sephadex LH-20 column chromatography (MeOH) and reversed phase RP-18 column chromatography with a stepwise gradient of MeOH-H_2_O (20:80→100:0) to provide ten fractions (Fr.1a-1j). Fraction 1e (37.6 mg) was further purified by PTLC with CH_2_Cl_2_-MeOH (98:2) to yield 8-*O*-methyltetrangomycin (**3**, 17.5 mg, *R*_f_ = 0.3, CH_2_Cl_2_-MeOH (95:5)), and fraction 1f (20 mg) was further purified by PTLC with CH_2_Cl_2_-MeOH (98:2) to yield kiamycin (**1**, 6.3 mg, *R*_f_ = 0.4, CH_2_Cl_2_-MeOH (95:5)) and 8-*O*-methylrabelomycin (**4**, 2.6 mg, *R*_f_ = 0.3, CH_2_Cl_2_-MeOH (95:5)). 

### 3.5. Cytotxicity Tests

Cytotxicity tests were carried out according to the method previously described by Zhang *et al.* [14] with slight modification: Kiamycin (**1**) solutions (2 μL in MeOH) were added to each well and further incubated for 72 h under the same conditions at the concentration of 10^−4^ M.

## 4. Conclusions

A new angucycline derivative, kiamycin (**1**), possessing an 1,12-epoxybenz[a]anthracene ring system, was isolated from the marine *Streptomyces* sp. M268. This is the first report of this skeleton from nature. The new compound **1** exhibited cytotoxic activities against three human cell lines, including leukemia HL-60, lung adenocarcinoma A549, and hepatoma cell line BEL-7402. 

## Supplementary Files

Supplementary File 1: ZIP-Document (ZIP, 602 KB)
