# Supplementary material for: Kiamycin, a Unique Cytotoxic Angucyclinone Derivative from a Marine Streptomyces sp
Source: Mar Drugs. 2012 Feb 27;10(3):551–8. doi: 10.3390/md10030551 (PMC3347014; doi:10.3390/md10030551)
Supplement: Supplementary File 1: — ZIP-Document (ZIP, 602 KB) [file marinedrugs-10-00551-s001.zip › marinedrugs-14562-supplementary-revised version 1/marinedrugs-14562-supplementary 1.pdf]

Laa

Name Xie, Zeping **Abteilung** Laa **Telefon** 6235 **Datum** 05.07.11

Bezeichnung der Substanz: ZX047

Schmelzpunkt bzw. Siedepunkt: $CDCl_3$

Lösungsmittel:

Empfindlich gegen:

Summenformel:

Molekulargewicht:

Exakte Masse

Achtung! Massenfeinbestimmungen sagen ***nichts*** über die Reinheit einer Verbindung aus !

$[M+Na]^+$ = 345.1

$[2M+Na]^+$ = 667.3

$[M-H]^-$ = 321.1

$[2M-H]^-$ = 643.2

Vermutliche Struktur: Natural product

Syntheseweg:

Umkristallisiert aus:

Sonstige Verunreinigungen:

Rückgewinnung der Substanz (ja – nein):

Elementaranalyse vorhanden (ja – nein); Begründung, wenn nicht vorhanden:

Bereits vorliegende spektroskopische Befunde:

Aufgabenstellung:

EI	ESI	DCI	MALDI	FAB	APCI*	GC-MS*	LC-MS*	HR-MS	MS/MS*
	X							X	

* nach Absprache

Display Report

Analysis Info

Analysis Name	Z:\Data\2011\1107Jul\sam070711\ZX047 low_18_01_35752.d	Acquisition Date	06.07.2011 23:01:57
Method	fiamsauto_pos_lowlow.m	Operator	BDAL@DE
Sample Name	ZX047 low	Instrument / Ser#	micrOTOF 10237
Comment			

Acquisition Parameter

Source Type	ESI	Ion Polarity	Positive	Set Nebulizer	1.6 Bar
Focus	Not active			Set Dry Heater	180 °C
Scan Begin	50 m/z	Set Capillary	4500 V	Set Dry Gas	8.0 l/min
Scan End	1600 m/z	Set End Plate Offset	-500 V	Set Divert Valve	Source

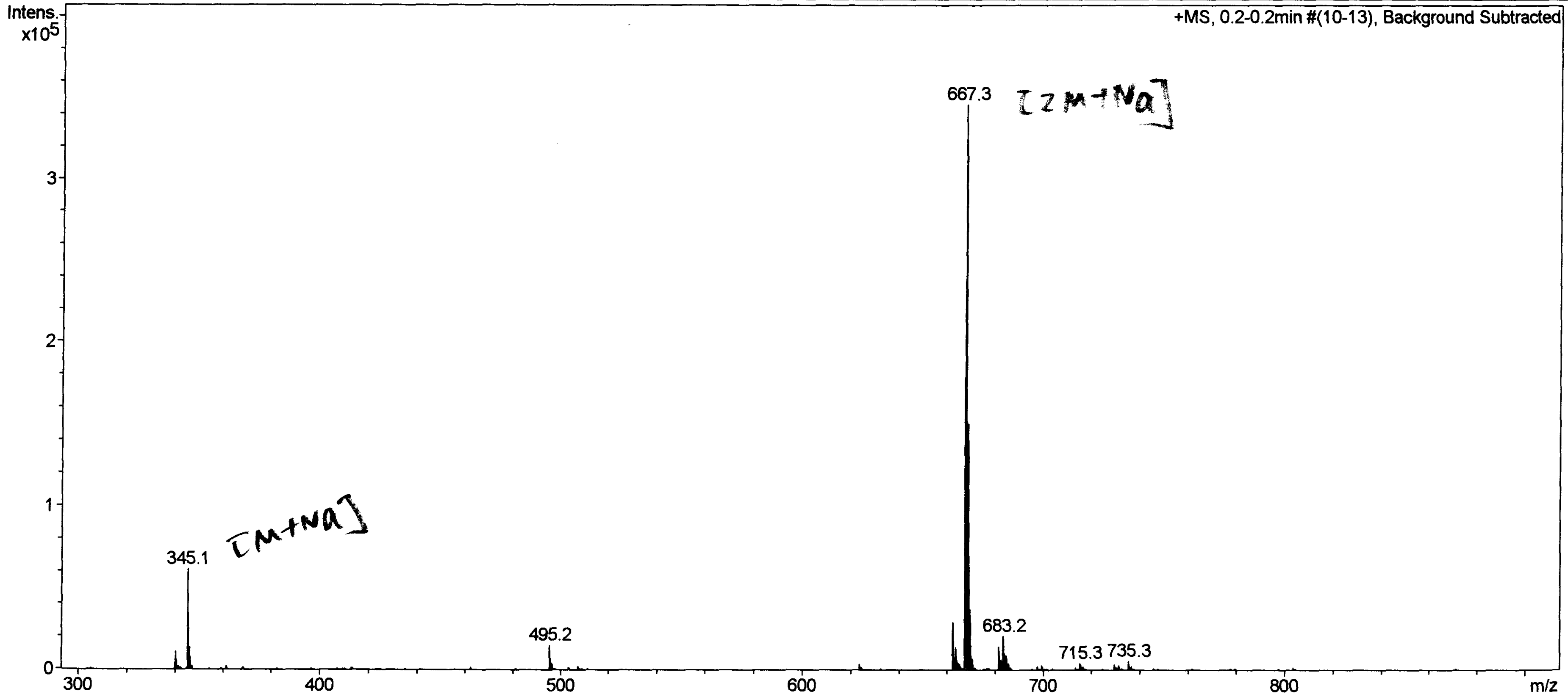

Mass Spectrum SmartFormula Report

Analysis Info

Analysis Name	Z:\Data\2011\1107Jul\sam070711\ZX047 low_18_01_35752.d	Acquisition Date	06.07.2011 23:01:57
Method	fiamsauto_pos_lowlow.m	Operator	BDAL@DE
Sample Name	ZX047 low	Instrument / Ser#	micrOTOF 10237
Comment			

Acquisition Parameter

Source Type	ESI	Ion Polarity	Positive	Set Nebulizer	1.6 Bar
Focus	Not active			Set Dry Heater	180 °C
Scan Begin	50 m/z	Set Capillary	4500 V	Set Dry Gas	8.0 l/min
Scan End	1600 m/z	Set End Plate Offset	-500 V	Set Divert Valve	Source

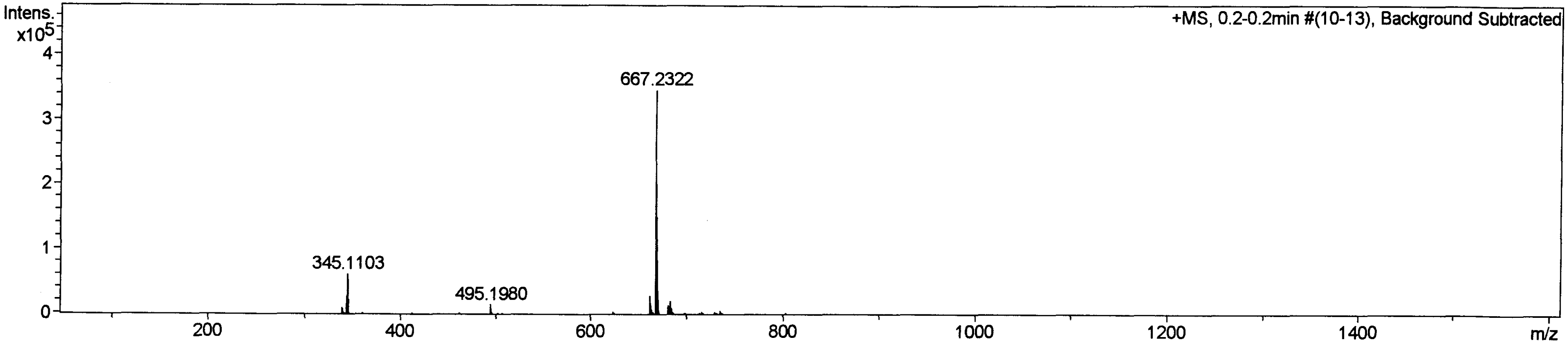

Meas. m/z	#	Formula	m/z	err [ppm]	Mean err [ppm]	rdb	N-Rule	e⁻ Conf	mSigma	Std I	Std Mean m/z	Std I VarNorm	Std m/z Diff	Std Comb Dev
345.1103														
	1	C 20 H 18 Na O 4	345.1097	-1.6	-1.3	11.5	ok	even	4.08	0.0069	0.0005	0.0029	0.0005	0.6676
	2	C 21 H 14 N 4 Na	345.1111	2.3	2.5	16.5	ok	even	11.00	0.0160	0.0009	0.0073	0.0004	0.8427
	3	C 16 H 14 N 6 Na O 2	345.1070	-9.4	-9.1	12.5	ok	even	17.15	0.0312	0.0032	0.0129	0.0004	0.9768

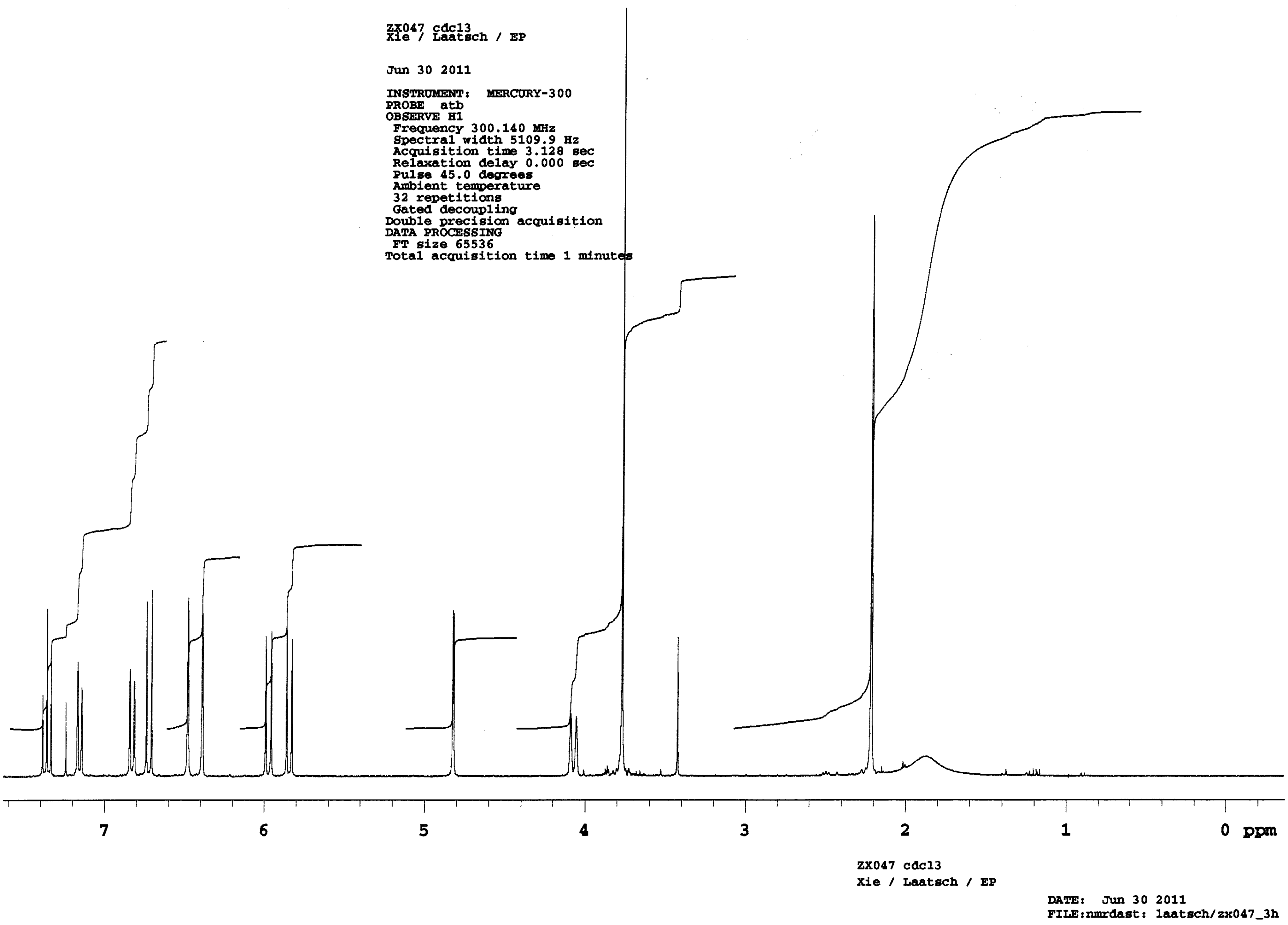
ZX047 cdcl3
Xie / Laatsch / EP
Jun 30 2011
INSTRUMENT: MERCURY-300
PROBE atb
OBSERVE H1
Frequency 300.140 MHz
Spectral width 5109.9 Hz
Acquisition time 3.128 sec
Relaxation delay 0.000 sec
Pulse 45.0 degrees
Ambient temperature
32 repetitions
Gated decoupling
Double precision acquisition
DATA PROCESSING
FT size 65536
Total acquisition time 1 minutes
7
6
5
4
3
2
1
0 ppm
ZX047 cdcl3
Xie / Laatsch / EP
DATE: Jun 30 2011
FILE:nmrdast: laatsch/zx047_3h

Display Report

Analysis Info

Analysis Name	Z:\Data\2011\1107Jul\sam070711\neg\ZX047 low_18_01_35725.d	Acquisition Date	06.07.2011 20:27:02
Method	fiamsauto_neg_lowlow.m	Operator	BDAL@DE
Sample Name	ZX047 low	Instrument / Ser#	micrOTOF 10237
Comment			

Acquisition Parameter

Source Type	ESI	Ion Polarity	Negative	Set Nebulizer	1.6 Bar
Focus	Not active			Set Dry Heater	180 °C
Scan Begin	50 m/z	Set Capillary	3800 V	Set Dry Gas	8.0 l/min
Scan End	1600 m/z	Set End Plate Offset	-500 V	Set Divert Valve	Source

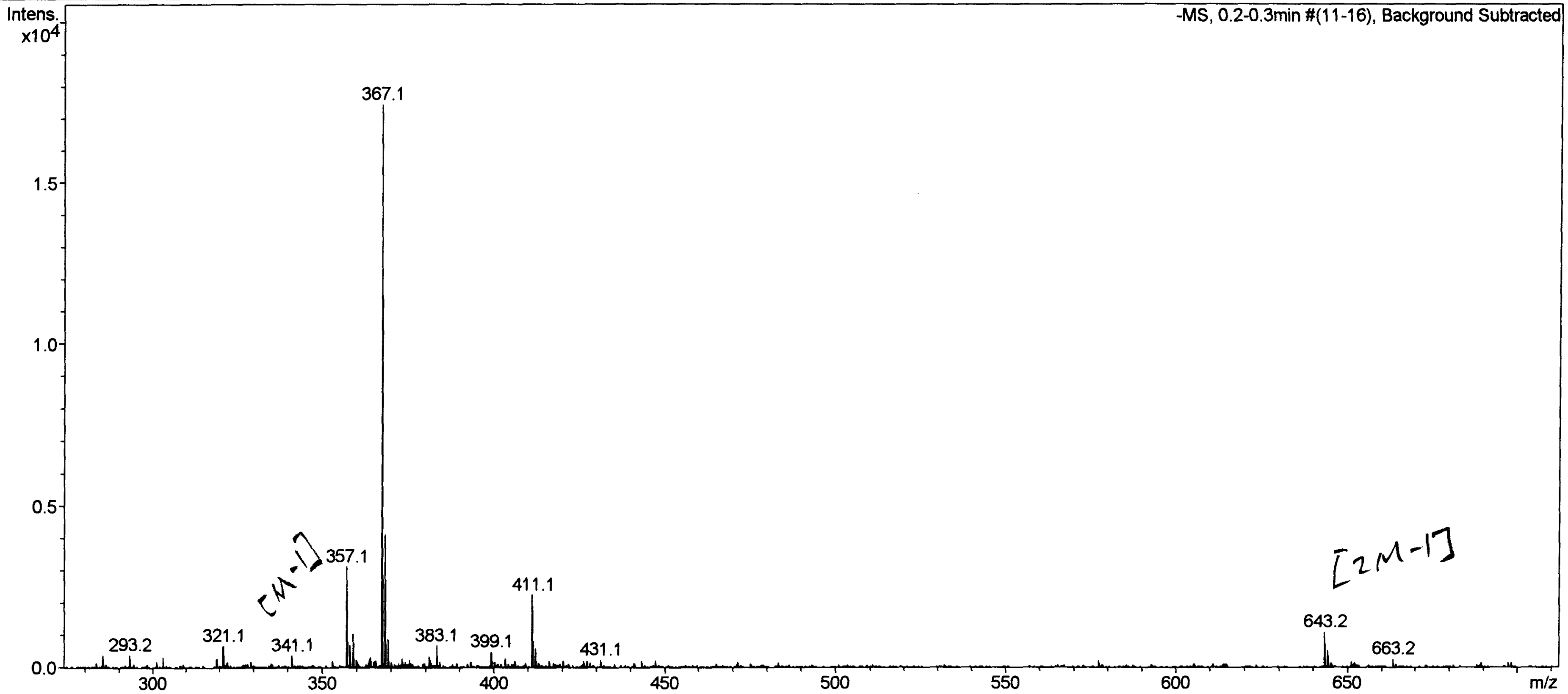

ZX047 cdcl3
Xie/Laatsch

Jul 10 2011

INSTRUMENT VNMRS-300
Pulse sequence gCOSY
OBSERVE H1
Frequency 300.535 MHz
Spectral width 2323.4 Hz
2D Spectral width 2323.4 Hz
Acquisition time 0.220 sec
Relaxation delay 1.000 sec
Ambient temperature
No. repetitions 1
No. increments 128
Double precision acquisition
DATA PROCESSING
Sine bell squared 0.110 sec
FT size 1024
F1 DATA PROCESSING
Sine bell square 0.055 sec
FT size 1024
Total acquisition time 3 minutes

VS= 2567

TH= 2

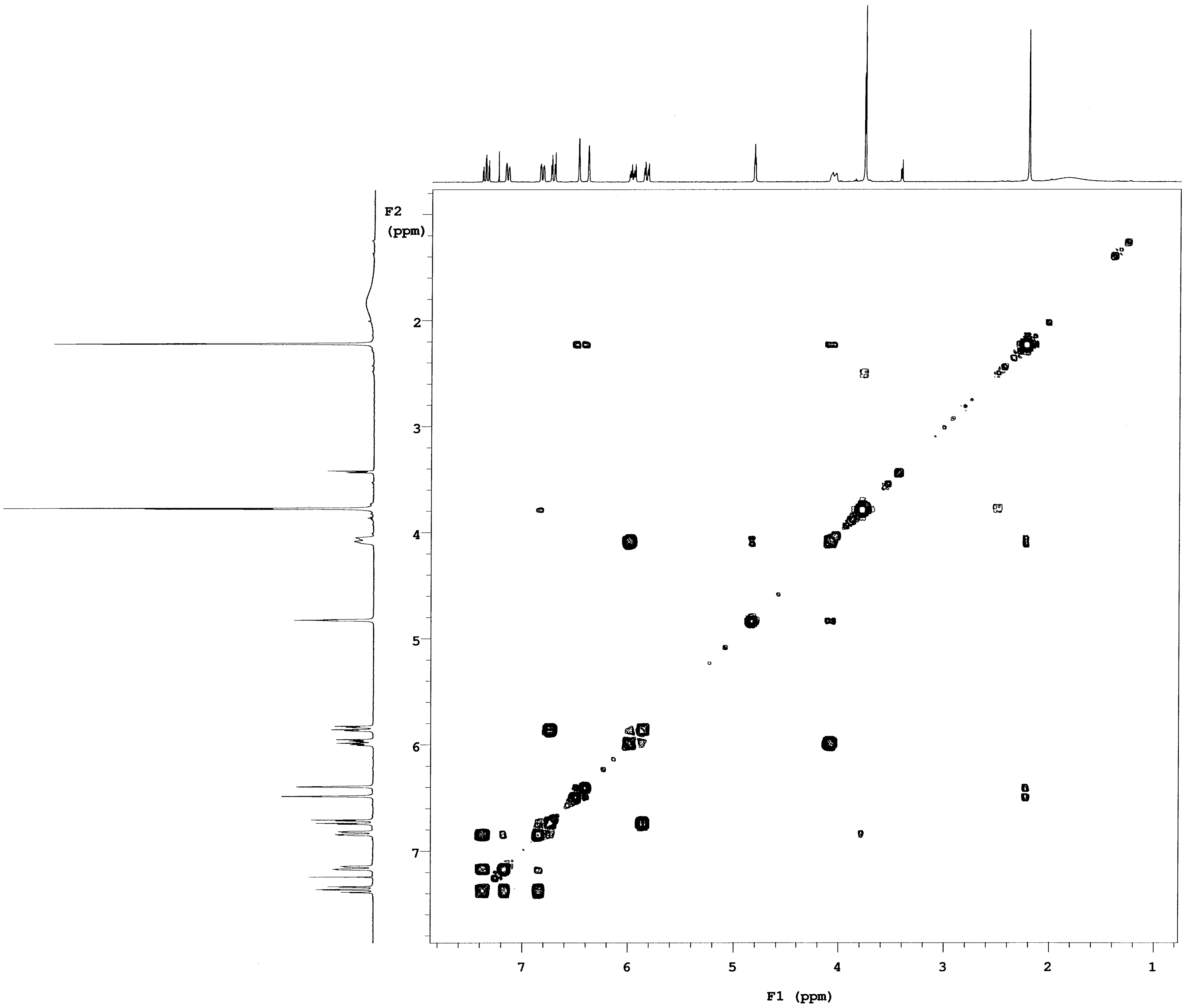

FILE=nmrdast: laatsch/zx047_3gcosy

ZX047 cdcl3
Xie/Laatsch

Jul 10 2011

INSTRUMENT VNMRS-300
Pulse sequence gHSQCAD
OBSERVE H1
Frequency 300.535 MHz
Spectral width 2323.4 Hz
2D Spectral width 12847.3 Hz
Acquisition time 0.230 sec
Relaxation delay 1.000 sec
Mixing time 0.500 sec
Ambient temperature
No. repetitions 8
No. increments 256 X2
DECOUPLE C13
Frequency 75.575 Mhz
Power 34 dB
Decoupler gated on during acquisition
Decoupler gated off during delay
W40_ATB_W005 modulated
Double precision acquisition
DATA PROCESSING
Gaussian apodization 0.106 sec
FT size 2048
F1 DATA PROCESSING
Gaussian apodization 0.018 sec
FT size 2048
Total acquisition time 1:59 hours

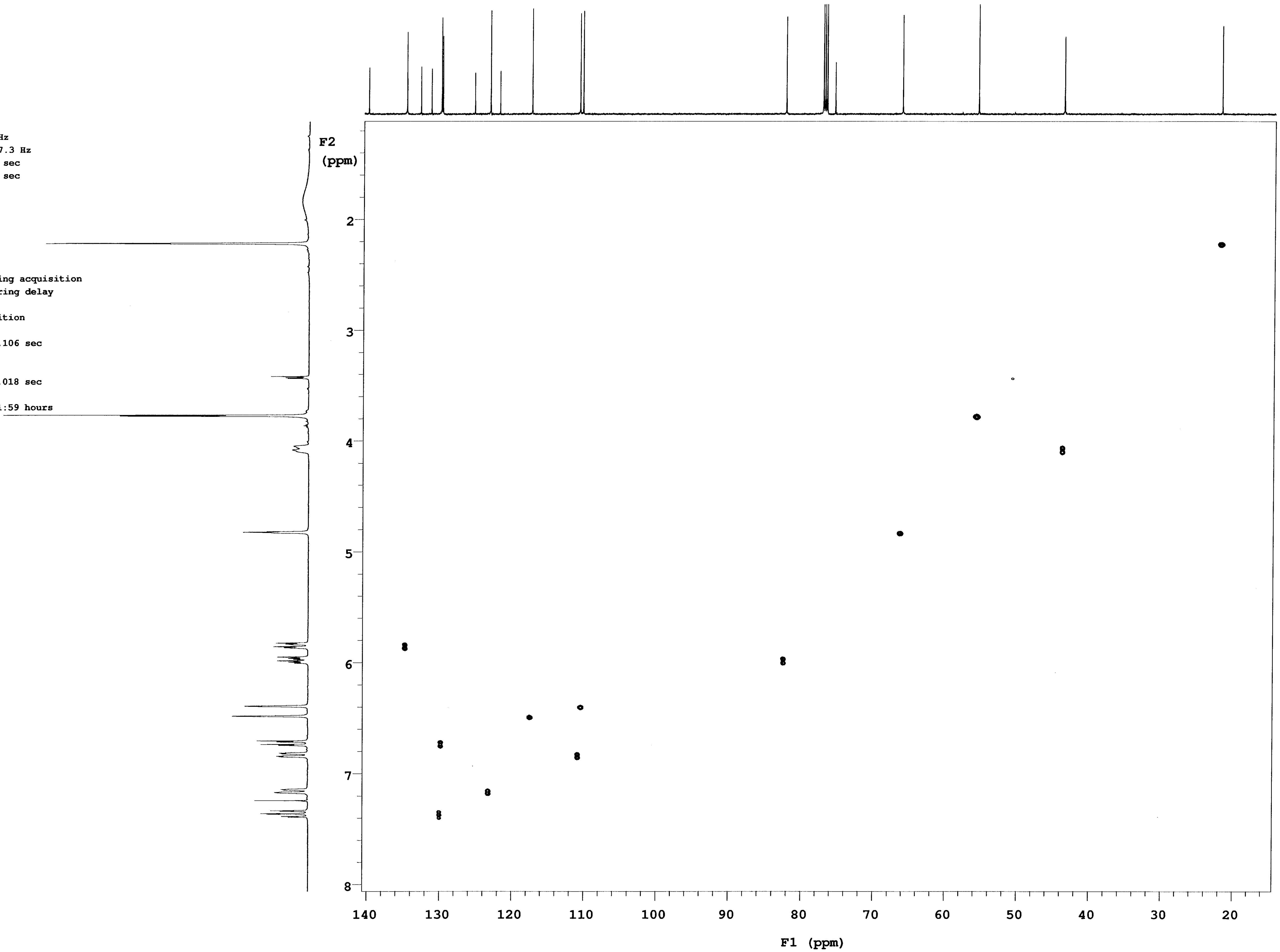

VS= 12147
TH= 5

FILE=

ZX047 cdcl3
Xie/Laatsch

Jul 10 2011

INSTRUMENT VNMRS-300
Pulse sequence gHSQCAD
OBSERVE H1
Frequency 300.535 MHz
Spectral width 2323.4 Hz
2D Spectral width 12847.3 Hz
Acquisition time 0.230 sec
Relaxation delay 1.000 sec
Mixing time 0.500 sec
Ambient temperature
No. repetitions 8
No. increments 256 X2
DECOUPLE C13
Frequency 75.575 Mhz
Power 34 dB
Decoupler gated on during acquisition
Decoupler gated off during delay
W40_ATB_W005 modulated
Double precision acquisition
DATA PROCESSING
Gaussian apodization 0.106 sec
FT size 2048
F1 DATA PROCESSING
Gaussian apodization 0.018 sec
FT size 2048
Total acquisition time 1:59 hours

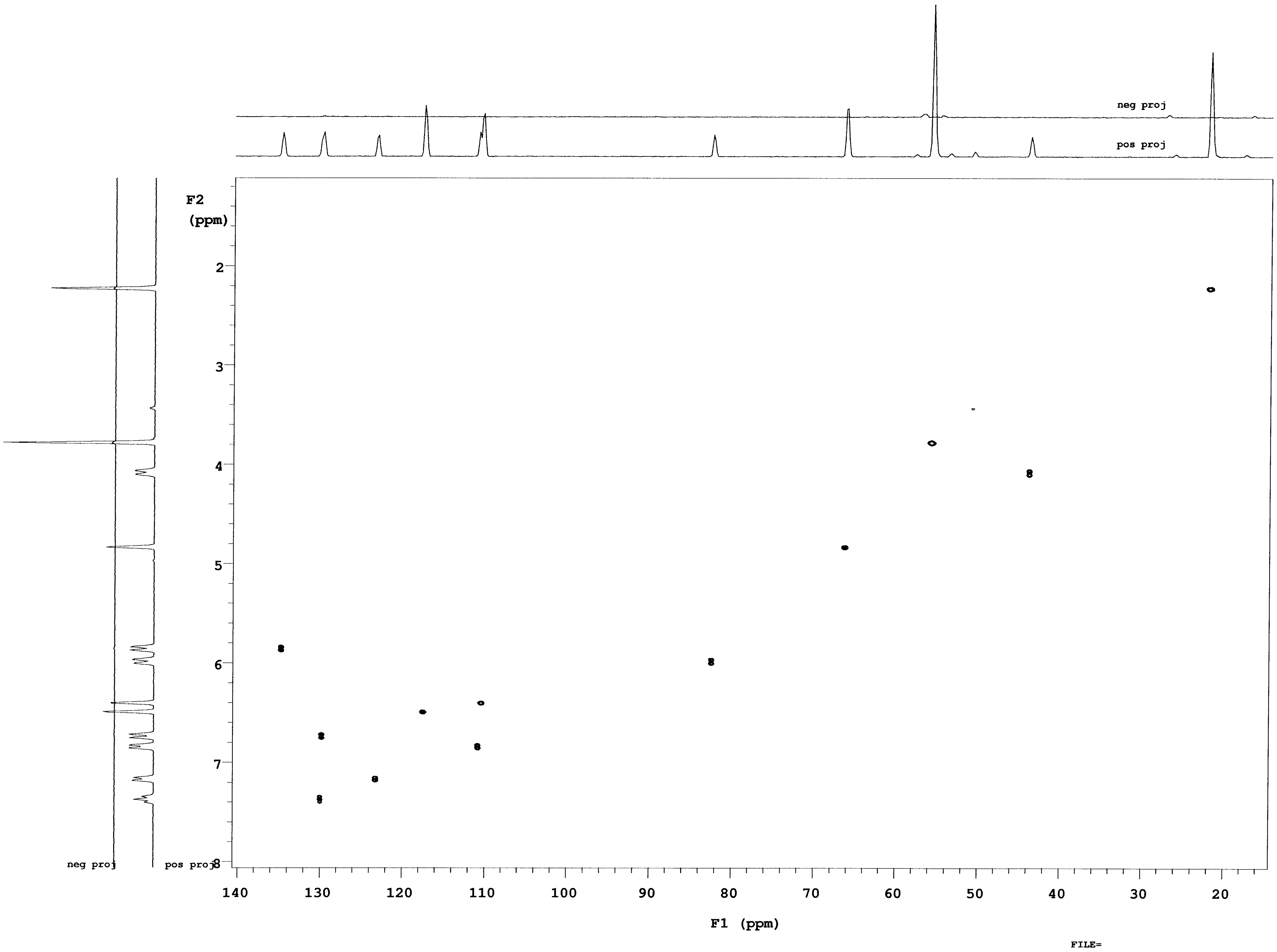

ZX047 cdcl3
Xie/Laatsch

Jul 10 2011

INSTRUMENT VNMRS-300
Pulse sequence gHMBC
OBSERVE H1
 Frequency 300.535 MHz
 Spectral width 2323.4 Hz
 2D Spectral width 12091.9 Hz
 Acquisition time 0.128 sec
 Relaxation delay 1.000 sec
 Mixing time 0.080 sec
 Ambient temperature
 No. repetitions 64
 No. increments 256
Double precision acquisition
DATA PROCESSING
 Sine bell 0.064 sec
 FT size 1024
F1 DATA PROCESSING
 Sine bell 0.021 sec
 FT size 2048
Total acquisition time 5:33 hours

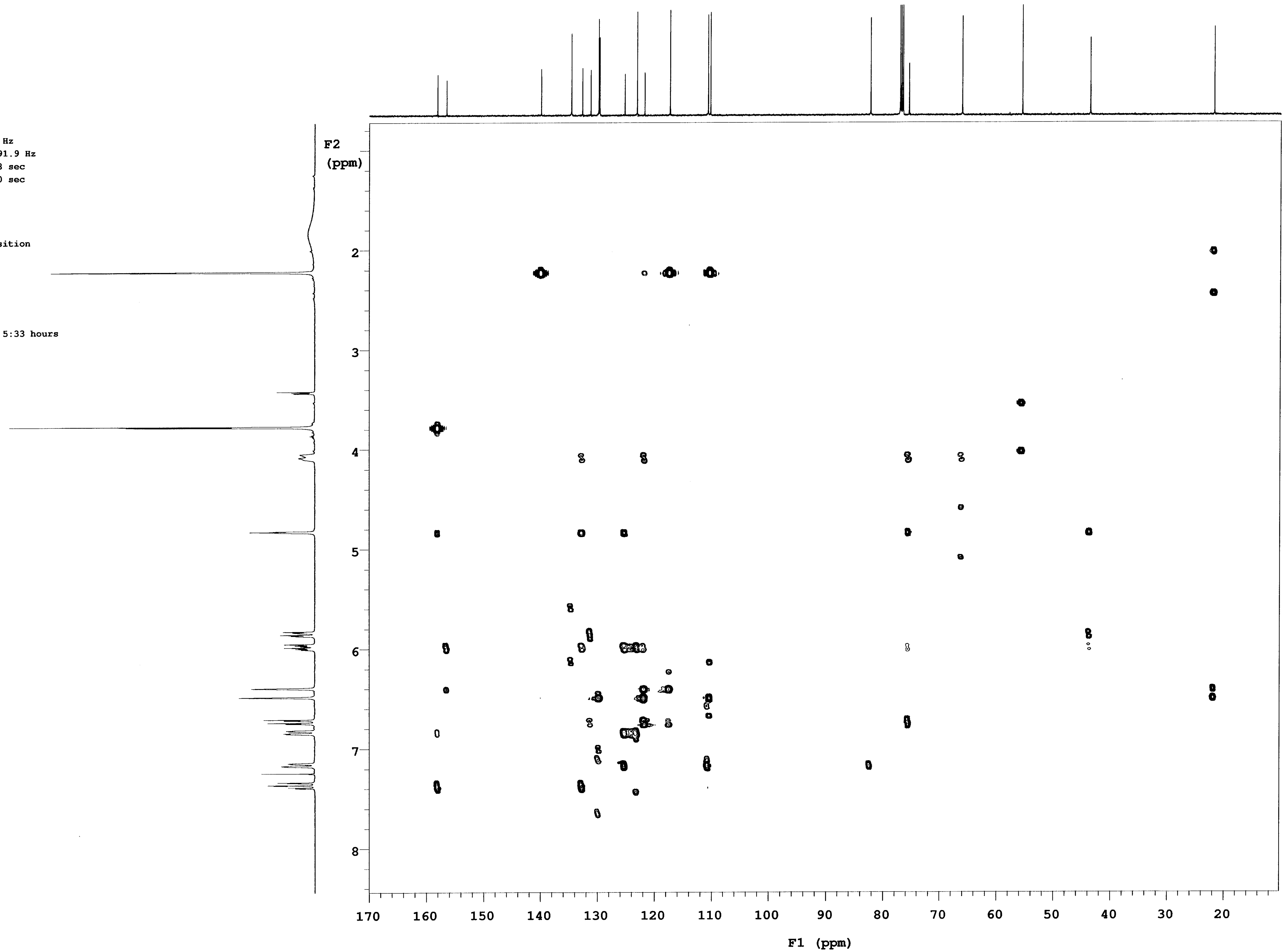

VS= 9201
TH= 2

FILE=nmrdast: laatsch/zx047_3ghmbc

ZX047 cdcl3
Xie/Laatsch

Jul 10 2011

INSTRUMENT VNMRS-300
Pulse sequence gHMBC
OBSERVE H1
Frequency 300.535 MHz
Spectral width 2323.4 Hz
2D Spectral width 12091.9 Hz
Acquisition time 0.128 sec
Relaxation delay 1.000 sec
Mixing time 0.080 sec
Ambient temperature
No. repetitions 64
No. increments 256
Double precision acquisition
DATA PROCESSING
Sine bell 0.064 sec
FT size 1024
F1 DATA PROCESSING
Sine bell 0.021 sec
FT size 2048
Total acquisition time 5:33 hours

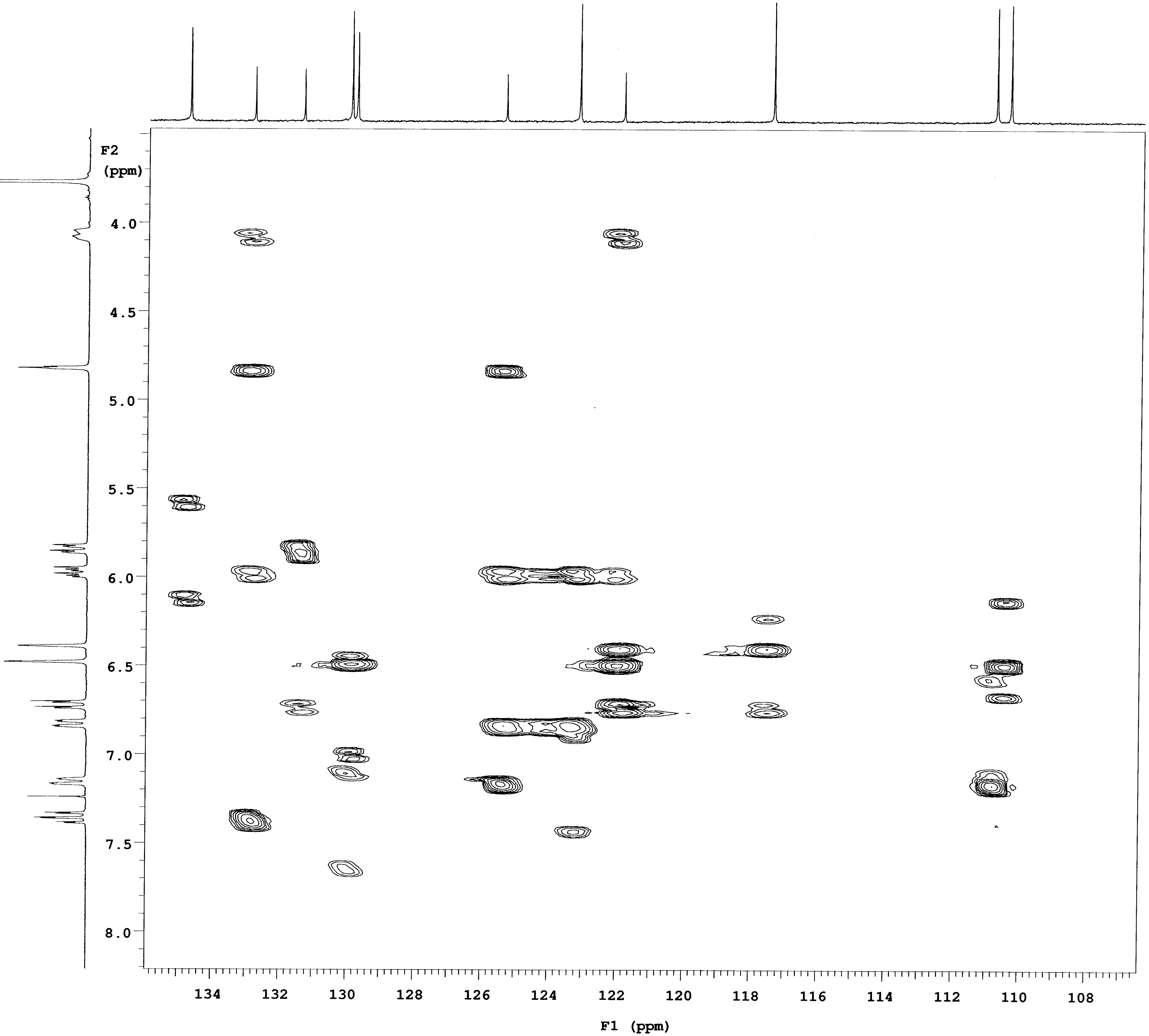

VS= 9201
TH= 2

FILE=nmrdast: laatsch/zx047_3ghmb[illegible]

ZX047 cdcl3
Xie/Laatsch

Jul 10 2011

INSTRUMENT VNMRS-300
Pulse sequence gHMBC
OBSERVE H1
Frequency 300.535 MHz
Spectral width 2323.4 Hz
2D Spectral width 12091.9 Hz
Acquisition time 0.128 sec
Relaxation delay 1.000 sec
Mixing time 0.080 sec
Ambient temperature
No. repetitions 64
No. increments 256
Double precision acquisition
DATA PROCESSING
Sine bell 0.064 sec
FT size 1024
F1 DATA PROCESSING
Sine bell 0.021 sec
FT size 2048
Total acquisition time 5:33 hours

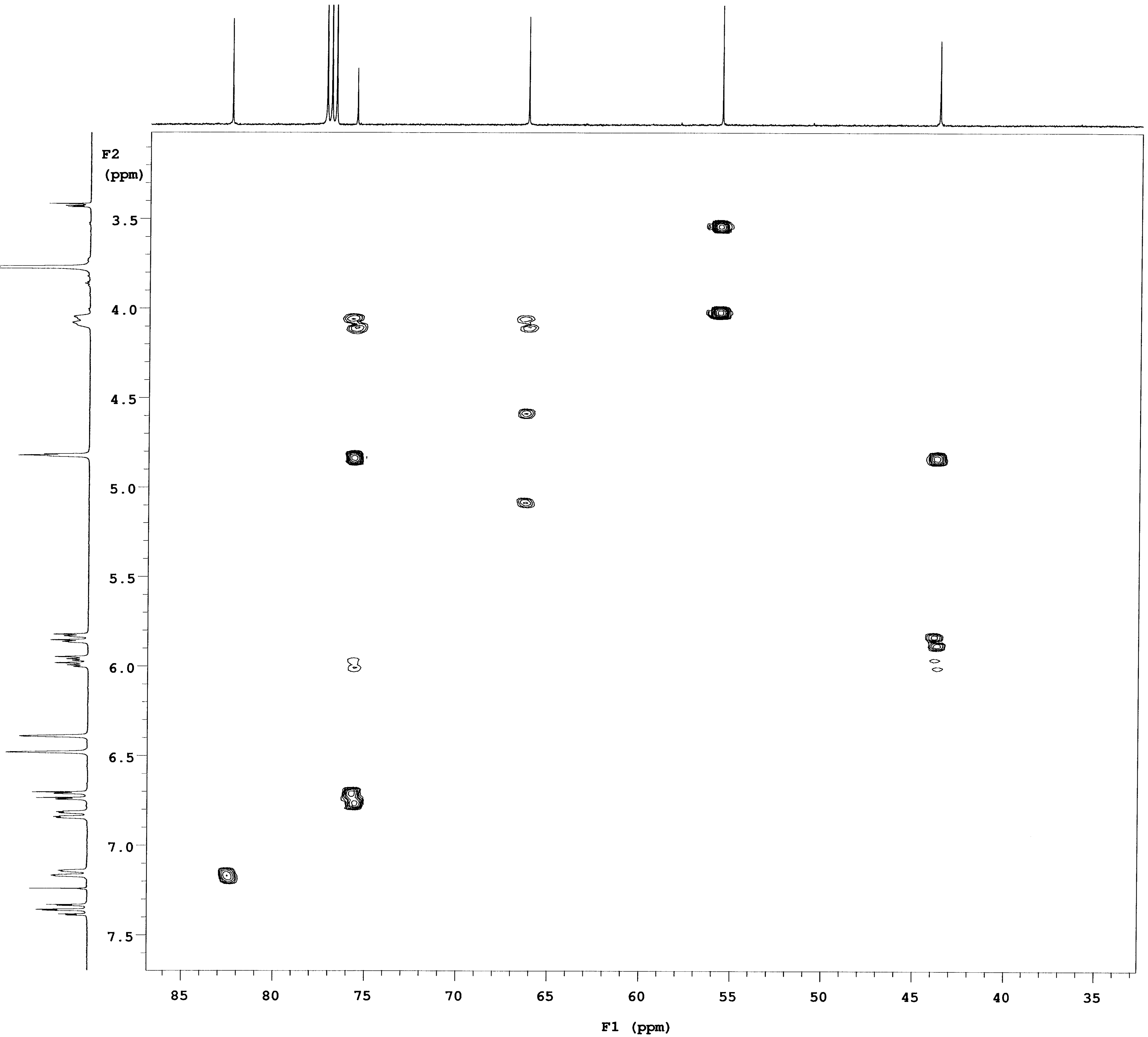

VS= 9201
TH= 2

FILE=nmrdast: laatsch/zx047_3ghmb[illegible]

zx047 cdcl3
Xie/Laatsch
Jul 5 2011

APT - Spectrum : CH,CH3 up / C,CH2 down

INSTRUMENT INOVA-500
PROBE coldc 3mm
Pulse sequence APT_ad
OBSERVE C13
Frequency 125.707 MHz
Spectral width 30165.9 Hz
Acquisition time 2.122 sec
Relaxation delay 2.000 sec
Pulse width 90.0 degrees
Temperature 27.0 deg. C / 300.1 K
No. repetitions 160
DECOUPLE H1
Frequency 499.877 Mhz
Power 32 dB
Decoupler gated on during acquisition
WALTZ-16 modulated
Double precision acquisition
DATA PROCESSING
Line broadening 1.0 Hz
FT size 262144
Total acquisition time 11 minutes

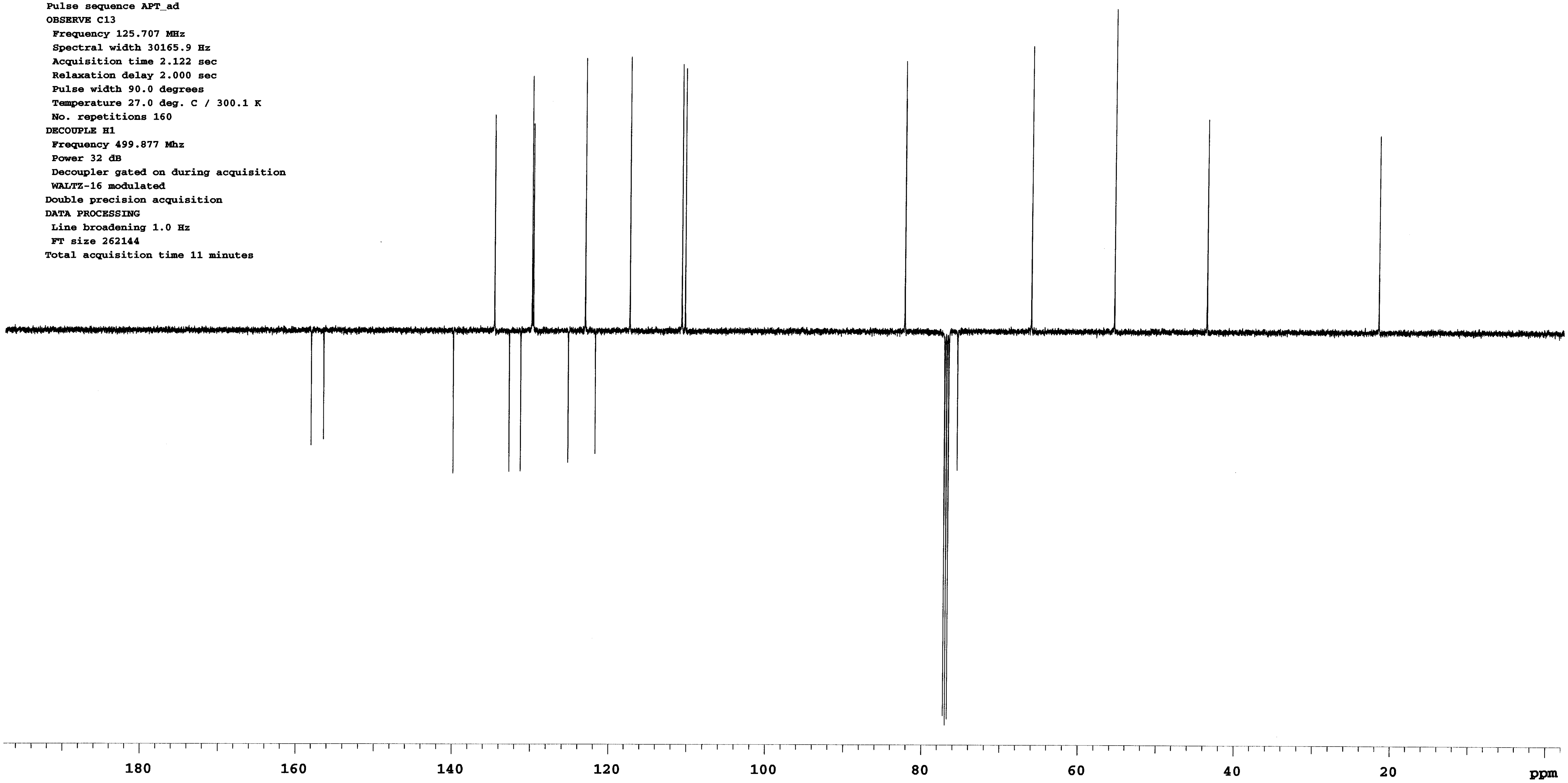

zx047 cdcl3
Xie/Laatsch

DATE: Jul 5 2011 FILE:nmrdast: laatsch/zx047_5apt_ad

zx047 cdcl3
Xie/Laatsch
Jul 5 2011

INSTRUMENT INOVA-500
PROBE coldc 3mm
OBSERVE C13
Frequency 125.707 MHz
Spectral width 30120.5 Hz
Acquisition time 2.125 sec
Relaxation delay 0.000 sec
Pulse width 36.0 degrees
Temperature 27.0 deg. C / 300.1 K
No. repetitions 640
DECOUPLE H1
Frequency 499.877 Mhz
Power 32 dB
Decoupler continuously on
WALTZ-16 modulated
Double precision acquisition
DATA PROCESSING
Line broadening 1.0 Hz
FT size 262144
Total acquisition time 22 minutes

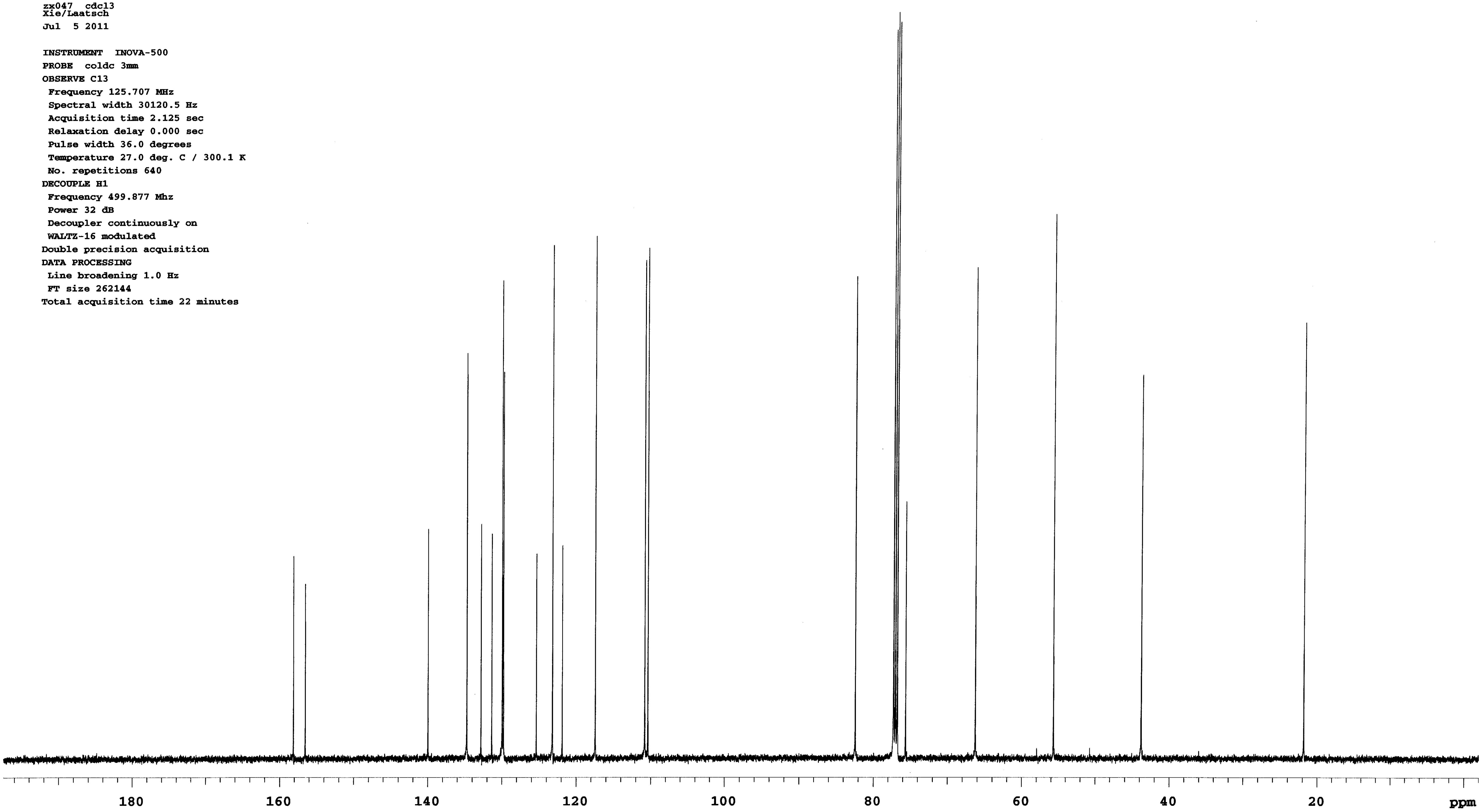

zx047 cdcl3
Xie/Laatsch

DATE: Jul 5 2011 FILE:nmrdast: laatsch/zx047_5c

zx047 cdcl3
Xie / Laatsch / mw

Sep 3 2011

INSTRUMENT INOVA-600
Pulse sequence NOESY
OBSERVE H1
Frequency 599.740 MHz
Spectral width 4845.0 Hz
2D Spectral width 4845.0 Hz
Acquisition time 0.211 sec
Relaxation delay 1.000 sec
Mixing time 1.000 sec
Ambient temperature
No. repetitions 16
No. increments 256 X2
Double precision acquisition
DATA PROCESSING
Gaussian apodization 0.098 sec
FT size 2048
F1 DATA PROCESSING
Gaussian apodization 0.049 sec
FT size 2048
Total acquisition time 5:06 hours

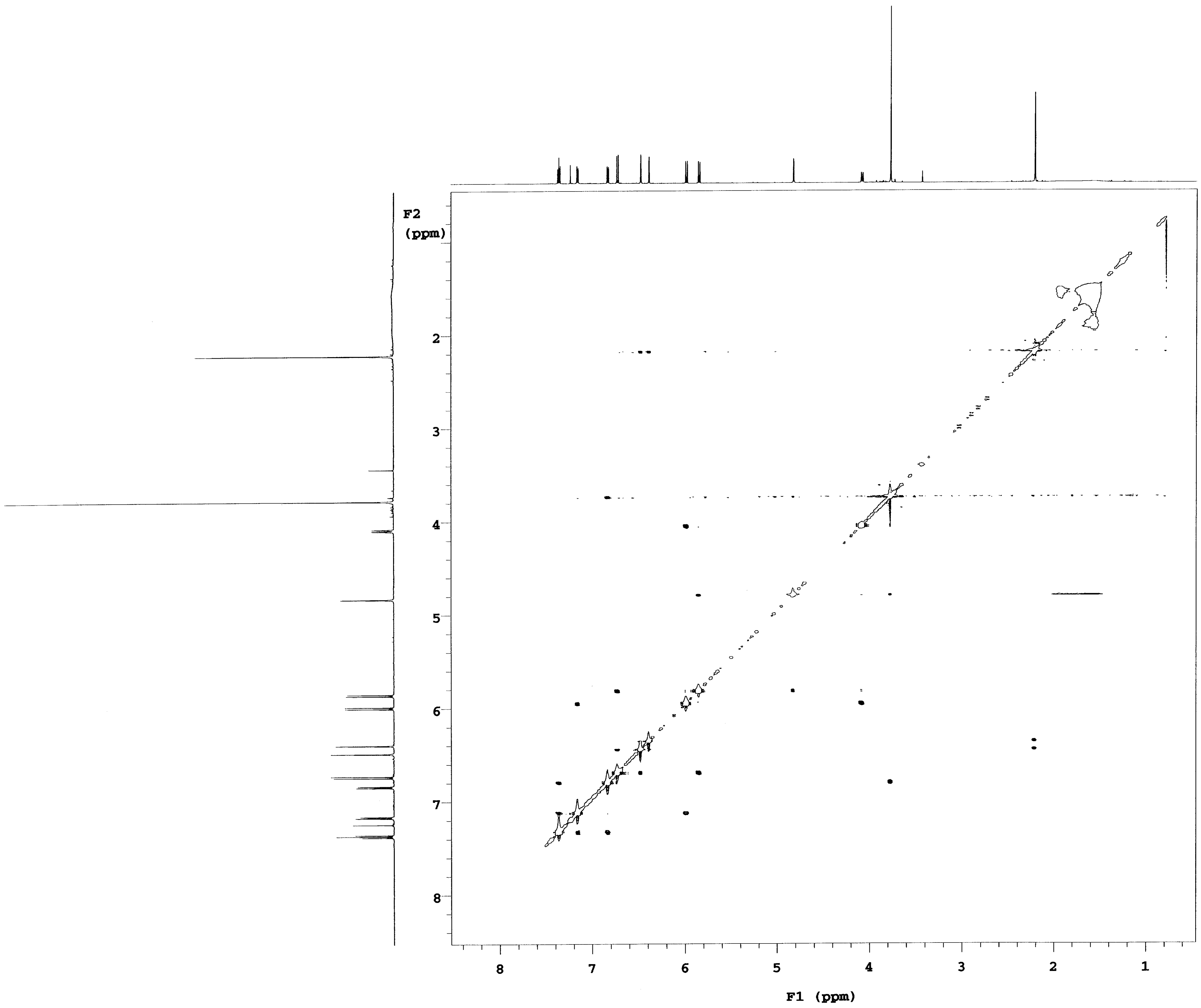

FILE=nmrdast: laatsch/zx047_6noesy

VS= 138
TH= 2
