# Supplementary material for: Kiamycin, a Unique Cytotoxic Angucyclinone Derivative from a Marine Streptomyces sp
Source: Mar Drugs. 2012 Feb 27;10(3):551–8. doi: 10.3390/md10030551 (PMC3347014; doi:10.3390/md10030551)
Supplement: Supplementary File 1: — ZIP-Document (ZIP, 602 KB) [file marinedrugs-10-00551-s001.zip › marinedrugs-14562-supplementary-revised version 1/Spectrum of 8-O-methylrabelomycin (4).pdf]

ZX046 cdcl3
Xie / Laatsch / EP

Jun 29 2011

INSTRUMENT: MERCURY-300
PROBE atb
OBSERVE H1
Frequency 300.140 MHz
Spectral width 5109.9 Hz
Acquisition time 3.128 sec
Relaxation delay 0.000 sec
Pulse 45.0 degrees
Ambient temperature
48 repetitions
Gated decoupling
Double precision acquisition
DATA PROCESSING
FT size 65536
Total acquisition time 2 minutes

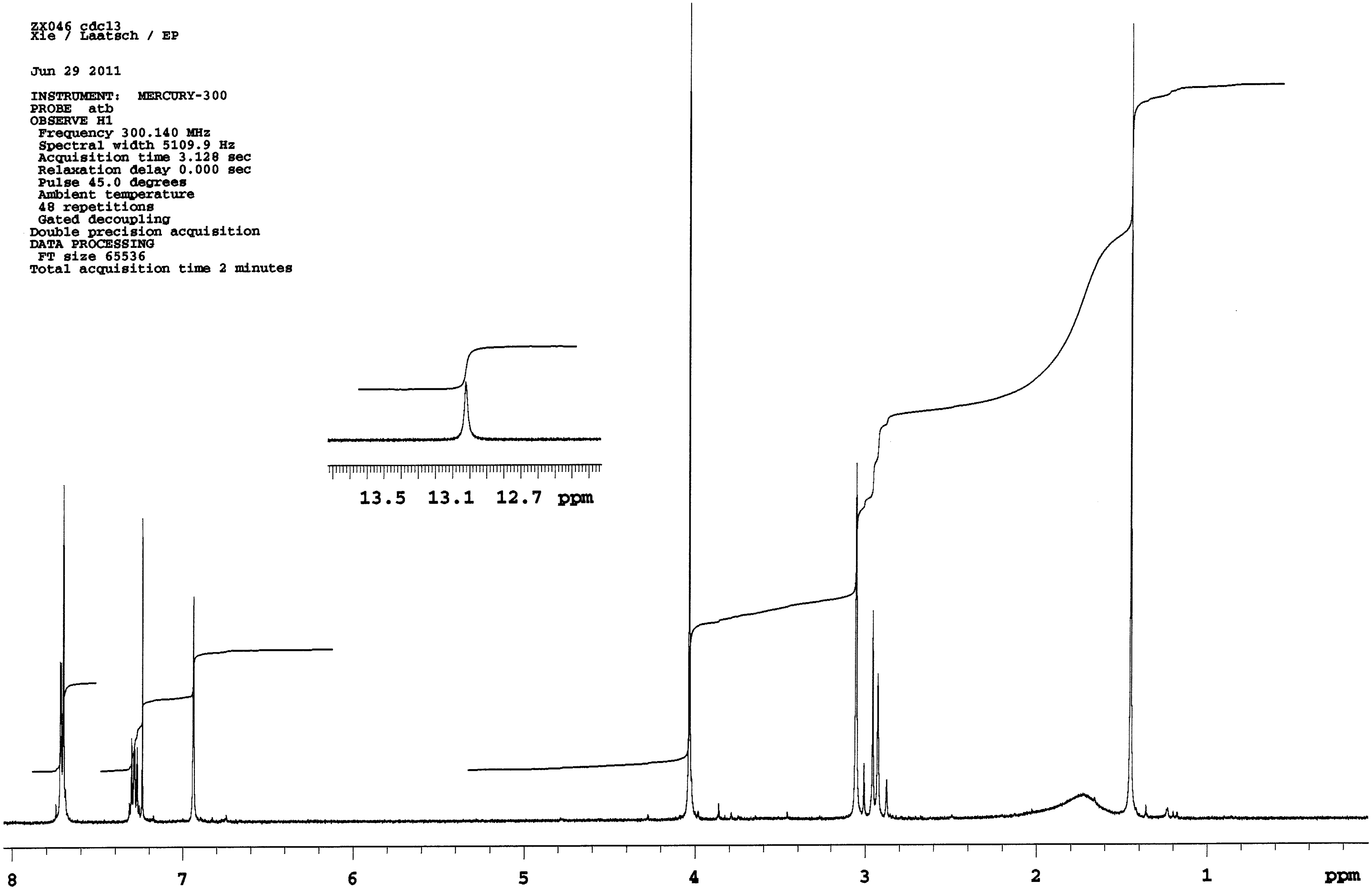

ZX046 cdcl3
Xie / Laatsch / EP

DATE: Jun 29 2011
FILE:nmrdast: laatsch/zx046_3h

zx046 cdcl3
Xie/Laatsch

SPECTRAL LINES FOR TH= 6.9
FROM 11.4 PPM TO 211.4 PPM
RFL= 11549.8 RFP= 9678.4

INDEX	FREQ	PPM	HEIGHT
1	24605.6	195.76	16.4
2	23665.0	188.28	14.0
3	23209.1	184.65	13.3
4	20600.2	163.89	21.1
5	20145.1	160.27	16.6
6	18783.8	149.44	19.3
7	17316.3	137.77	17.0
8	17298.8	137.63	12.8
9	17140.0	136.36	37.2
10	15965.1	127.02	13.5
11	15309.7	121.80	35.3
12	15069.1	119.89	38.0
13	15037.8	119.64	11.2
14	14799.1	117.74	12.3
15	14755.4	117.39	36.6
16	9710.3	77.25	195.6
17	9678.4	77.00	200.0
18	9646.5	76.75	198.1
19	9051.3	72.01	30.1
20	7112.0	56.58	37.8
21	6752.1	53.72	38.9
22	5547.5	44.14	38.7
23	3743.8	29.79	38.2

zx046 cdcl3
Xie/Laatsch
Jul 5 2011

INSTRUMENT INOVA-500
PROBE coldc 3mm
OBSERVE C13
Frequency 125.707 MHz
Spectral width 30120.5 Hz
Acquisition time 2.125 sec
Relaxation delay 0.000 sec
Pulse width 36.0 degrees
Temperature 27.0 deg. C / 300.1 K
No. repetitions 464
DECOUPLE H1
Frequency 499.877 Mhz
Power 32 dB
Decoupler continuously on
WALTZ-16 modulated
Double precision acquisition
DATA PROCESSING
Line broadening 1.0 Hz
FT size 262144
Total acquisition time 16 minutes

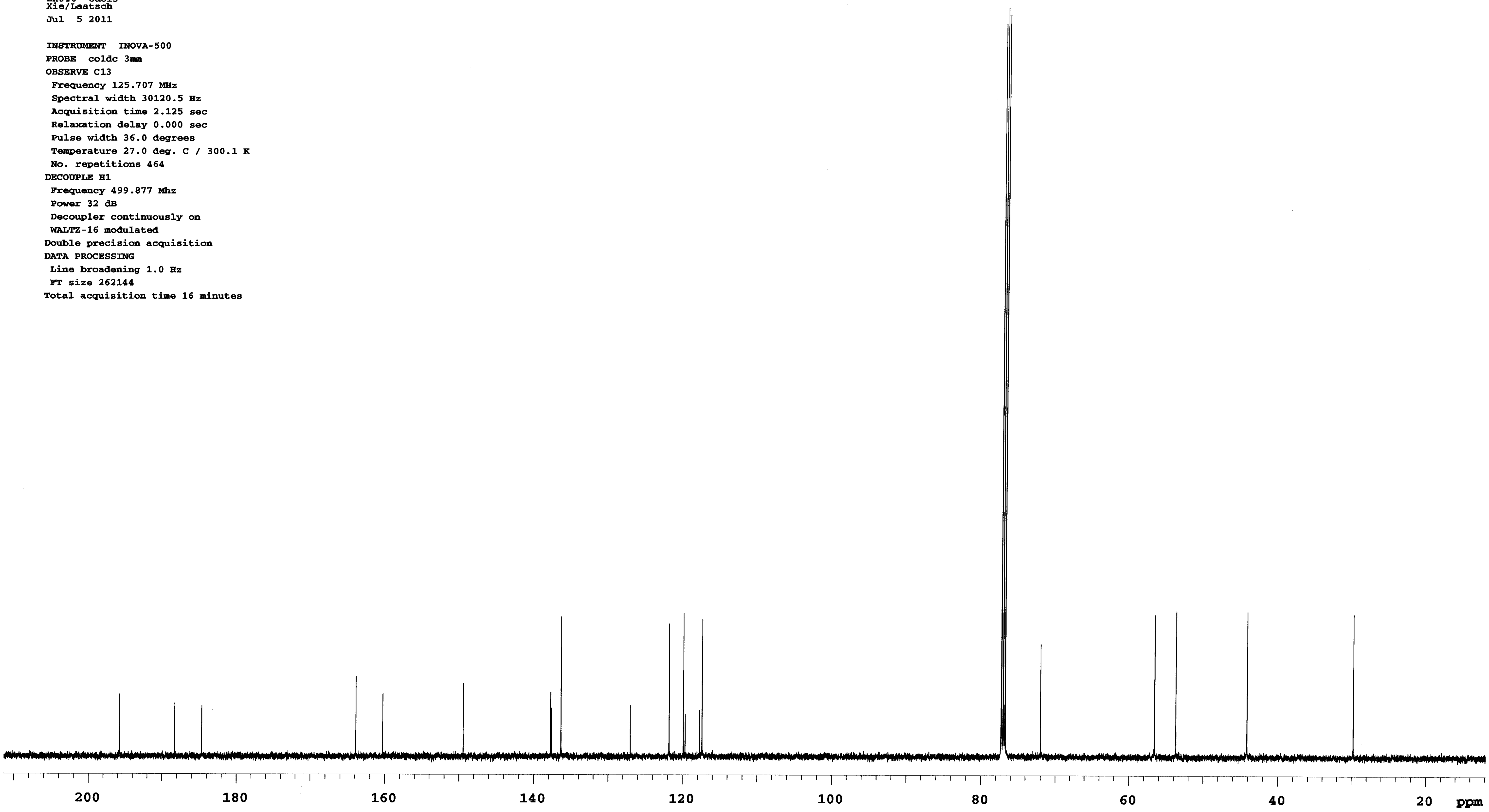

zx046 cdcl3
Xie/Laatsch

DATE: Jul 5 2011 FILE:nmrdast: laatsch/zx046_5c

zx046 cdcl3
Xie/Laatsch
Jul 5 2011

APT - Spectrum : CH,CH3 up / C,CH2 down

INSTRUMENT INOVA-500
PROBE coldc 3mm
Pulse sequence APT_ad
OBSERVE C13
 Frequency 125.707 MHz
 Spectral width 30165.9 Hz
 Acquisition time 2.122 sec
 Relaxation delay 2.000 sec
 Pulse width 90.0 degrees
 Temperature 27.0 deg. C / 300.1 K
 No. repetitions 112
DECOUPLE H1
 Frequency 499.877 Mhz
 Power 32 dB
 Decoupler gated on during acquisition
 WALTZ-16 modulated
Double precision acquisition
DATA PROCESSING
 Line broadening 1.0 Hz
 FT size 262144
Total acquisition time 8 minutes

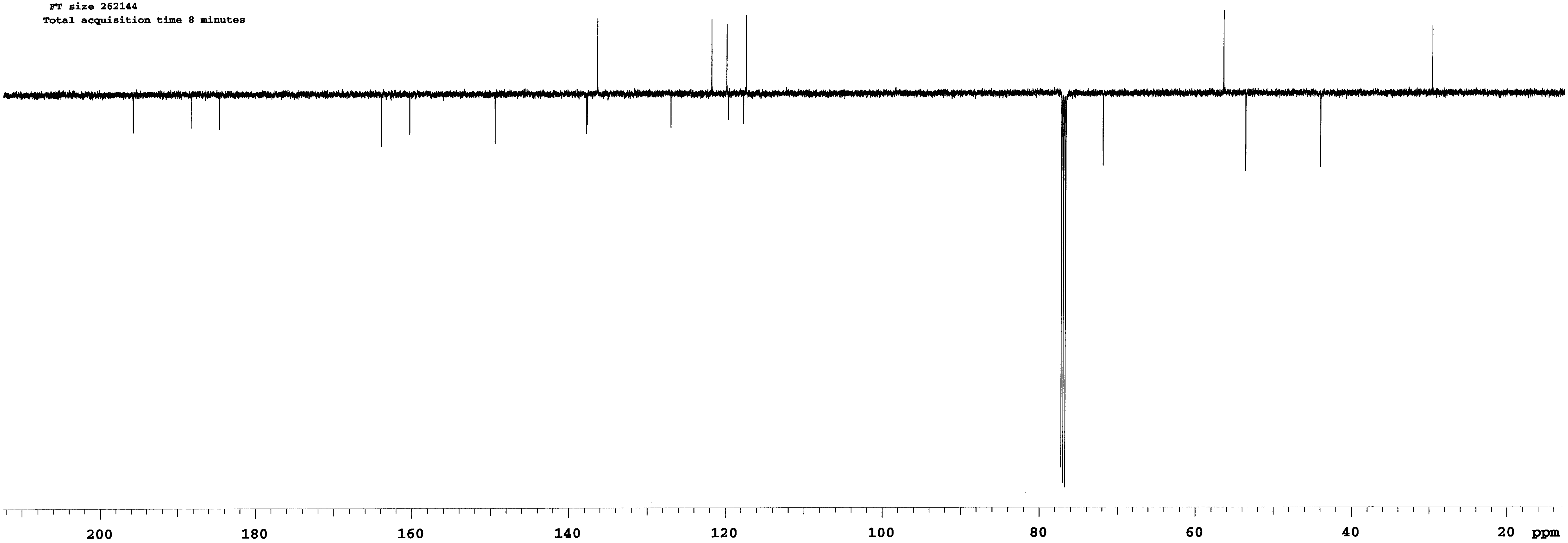

zx046 cdcl3
Xie/Laatsch

DATE: Jul 5 2011 FILE:nmrdast: laatsch/zx046_5apt_ad
