# Supplementary material for: Kiamycin, a Unique Cytotoxic Angucyclinone Derivative from a Marine Streptomyces sp
Source: Mar Drugs. 2012 Feb 27;10(3):551–8. doi: 10.3390/md10030551 (PMC3347014; doi:10.3390/md10030551)
Supplement: Supplementary File 1: — ZIP-Document (ZIP, 602 KB) [file marinedrugs-10-00551-s001.zip › marinedrugs-14562-supplementary-revised version 1/Spectrum of 8-O-methyltetrangomycin (3).pdf]

ZX045 cdcl3
Xie / Laatsch / EP

Jun 29 2011

INSTRUMENT: MERCURY-300
PROBE atb
OBSERVE H1
Frequency 300.140 MHz
Spectral width 5109.9 Hz
Acquisition time 3.128 sec
Relaxation delay 0.000 sec
Pulse 45.0 degrees
Ambient temperature
32 repetitions
Gated decoupling
Double precision acquisition
DATA PROCESSING
FT size 65536
Total acquisition time 1 minutes

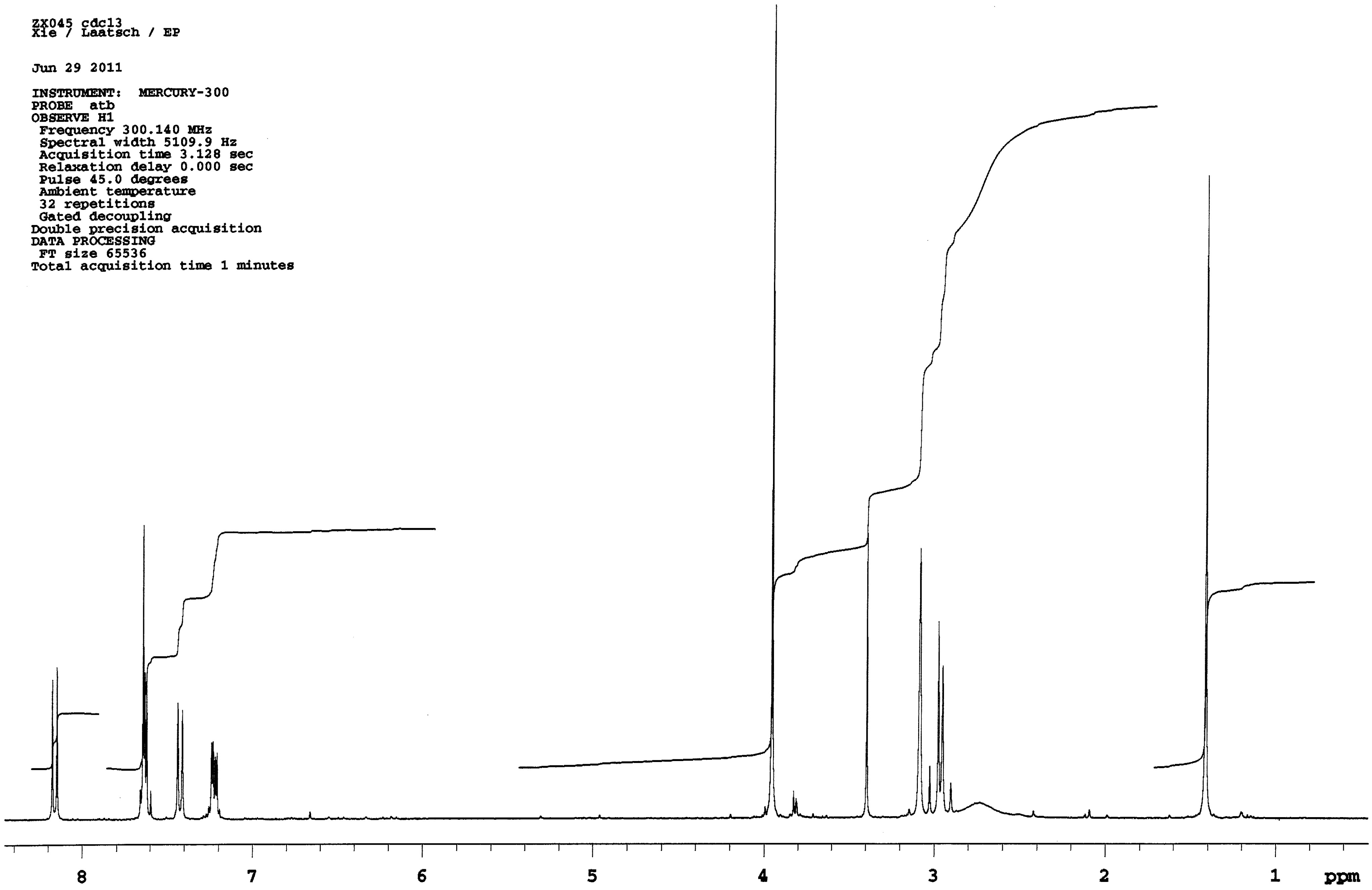

ZX045 cdcl3
Xie / Laatsch / EP

DATE: Jun 29 2011
FILE:nmrdast: laatsch/zx045_3h

ZX045 cdcl3
Xie / Laatsch / CS

SPECTRAL LINES FOR TH= 7.9
FROM 17.7 PPM TO 217.7 PPM
RFL= 11558.5 RFP= 9678.4

INDEX	FREQ	PPM	HEIGHT
1	24785.3	197.19	85.4
2	23197.6	184.56	71.6
3	22784.2	181.27	70.9
4	20071.8	159.69	90.1
5	18431.3	146.64	100.1
6	17287.1	137.53	83.7
7	17011.6	135.34	171.6
8	16966.3	134.98	71.7
9	16949.8	134.85	81.6
10	16851.6	134.07	79.6
11	16807.7	133.72	171.4
12	16324.5	129.88	166.5
13	15128.1	120.36	64.0
14	15014.6	119.45	175.3
15	14724.1	117.14	170.3
16	9710.3	77.25	181.8
17	9678.4	77.00	186.7
18	9646.5	76.75	183.8
19	9083.4	72.27	170.3
20	7086.0	56.38	200.0
21	6734.4	53.58	178.6
22	6352.5	50.54	24.6
23	5513.9	43.87	174.9
24	3735.1	29.72	188.4

ZX045 cdcl3
Xie / Laatsch / CS
Jun 30 2011

INSTRUMENT INOVA-500
PROBE coldc 3mm
OBSERVE C13
Frequency 125.707 MHz
Spectral width 30120.5 Hz
Acquisition time 2.125 sec
Relaxation delay 0.000 sec
Pulse width 36.0 degrees
Temperature 27.0 deg. C / 300.1 K
No. repetitions 640
DECOUPLE H1
Frequency 499.877 Mhz
Power 32 dB
Decoupler continuously on
WALTZ-16 modulated
Double precision acquisition
DATA PROCESSING
Line broadening 1.0 Hz
FT size 262144
Total acquisition time 22 minutes

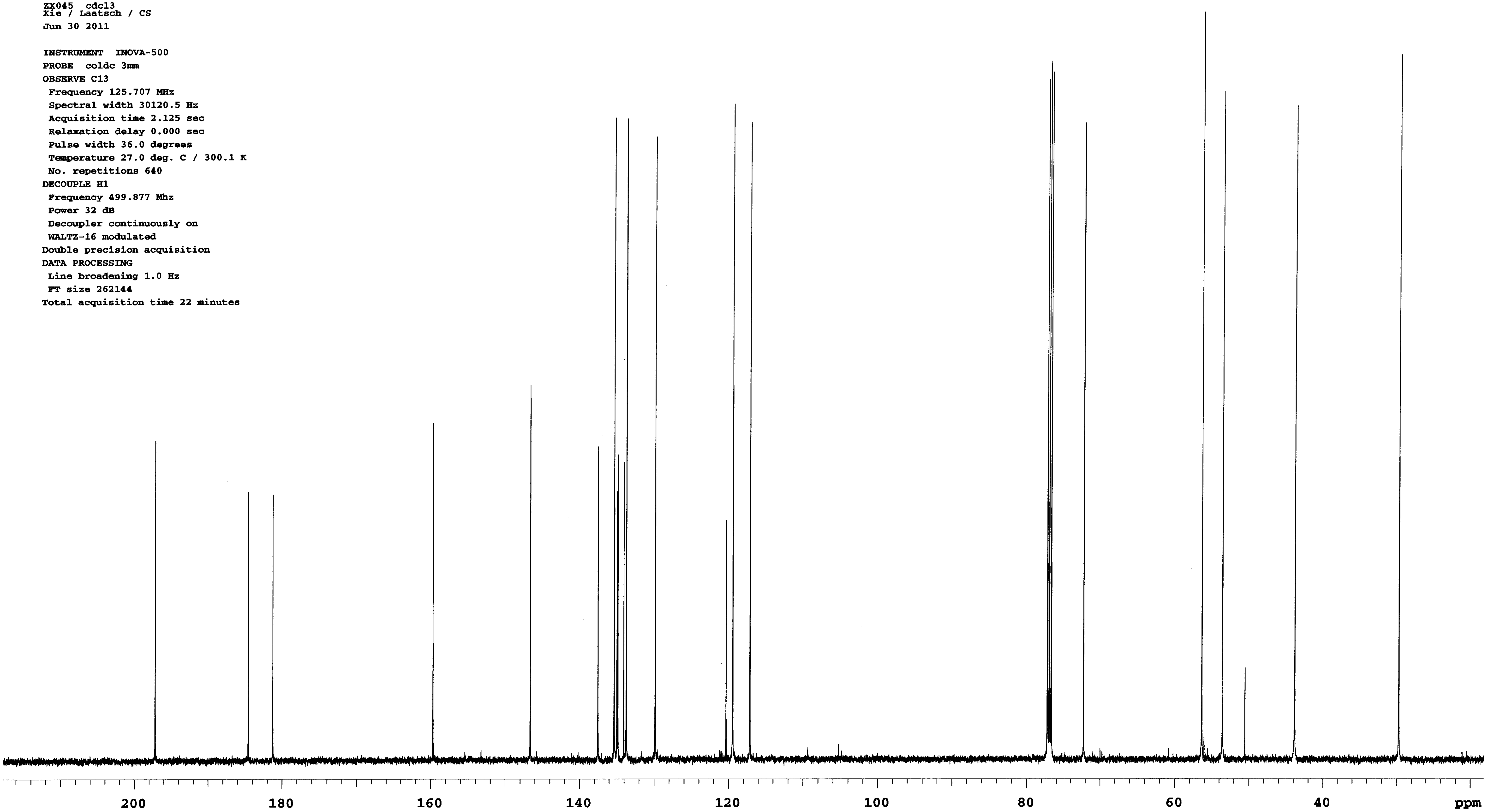

ZX045 cdcl3
Xie / Laatsch / CS

DATE: Jun 30 2011 FILE:nmrdast: laatsch/zx045_5c

ZX045 cdcl3
Xie / Laatsch / CS
Jun 30 2011

INSTRUMENT INOVA-500
PROBE coldc 3mm
Pulse sequence APT_ad
OBSERVE C13
Frequency 125.707 MHz
Spectral width 30165.9 Hz
Acquisition time 2.122 sec
Relaxation delay 2.000 sec
Pulse width 90.0 degrees
Temperature 27.0 deg. C / 300.1 K
No. repetitions 160
DECOUPLE H1
Frequency 499.877 Mhz
Power 32 dB
Decoupler gated on during acquisition
WALTZ-16 modulated
Double precision acquisition
DATA PROCESSING
Line broadening 1.0 Hz
FT size 262144
Total acquisition time 11 minutes

APT - Spectrum : CH,CH3 up / C,CH2 down

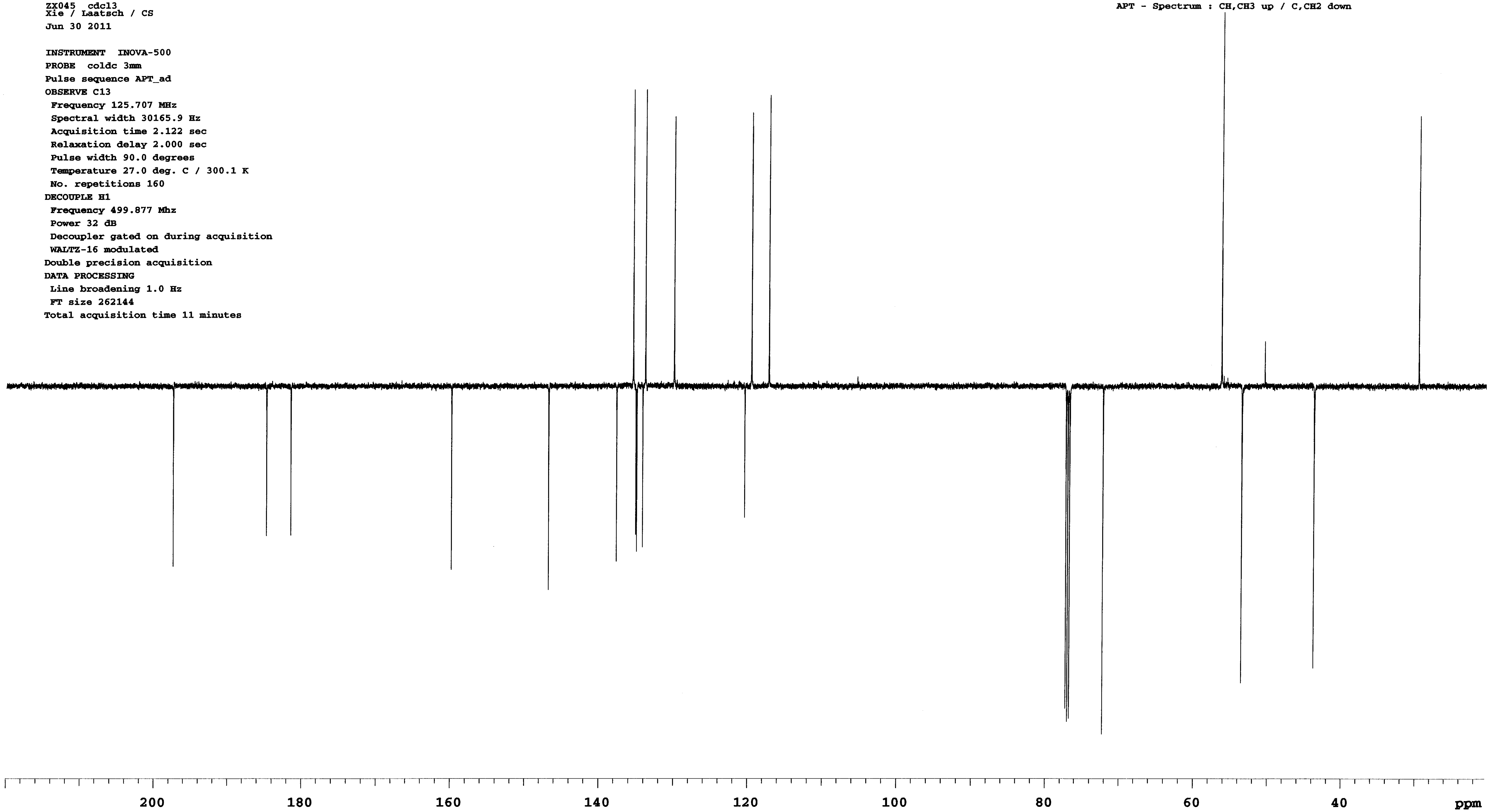

ZX045 cdcl3
Xie / Laatsch / CS

DATE: Jun 30 2011 FILE:nmrdast: laatsch/zx045_5apt_ad
